# Agro-industrial accidents linked to length of service, operation site and confidence in employer adherence to safety rules

**DOI:** 10.1186/s12889-020-08733-2

**Published:** 2020-04-30

**Authors:** Emmanuel Tamba Koroma, Jia Bainga Kangbai

**Affiliations:** 1 Department of Environmental Health Sciences, Njala University, Freetown, Sierra Leone; 2 Center for International Health, University of Munich (LMU), Munich, Germany

**Keywords:** Occupational health, Hazard, occupational accident, Occupational safety and health

## Abstract

**Background:**

The agriculture sector consistently ranks among the most hazardous occupational industries globally with high risk of job-related injuries, illnesses, disability, and death. In 2015, the agricultural fatal work injury rate in the United States was 22.8 per 100,000 full-time equivalent workers; seven times the all-worker fatal injury rate of 3.4 per 100,000 full-time equivalent workers. In this study we identified the factors that are associated with workplace accident and injuries at the Goldtree (SL) Limited Company - a private international agro-industrial palm kernel oil company operating in eastern Sierra Leone.

**Methods:**

This is a descriptive research that made use of both qualitative and quantitative research techniques to collect and analyse agro-industrial occupation-related accident and injuries of workers attached to the Goldtree (SL) Limited Company, an international palm kernel oil producing and marketing company in Sierra Leone. We analyzed the responses of 100 workers at the Goldtree (SL) Limited Company that are related to their work safety, adherence to work safety guidelines as well as working habit.

**Results:**

Thirty nine (39.0%) of the workers interviewed in this study said they had been involved in some forms of occupational accident; (33.3%) of those involved in some form of occupational-related accidents have been working in the company for 3–5 years, 22.0% have been working at the company for at least 2 years; 7.7% have been working for 6–8 years (X^2^ = 9.88, *p*-value = 0.02).

**Conclusion:**

Workers who have spent few years in the job, and those workers who have confidence that management is committed to addressing health and safety issues, believed that their working tools were in excellent condition, or agreed that they have the rights and responsibilities for an effective workplace health and safety system have decreased odds of experiencing occupational-related accidents or injury at the study site.

## Background

In spite of being one of the most important sectors globally in terms of food supply and workforce, the agro-industry is considered as one of the most hazardous sectors in terms of its high work-related illnesses, accident and mortality rates [1, 2]. The Occupational Safety and Health (OSH) management system which was developed by the International Labour Organization (ILO) seeks to address amongst many issues to recognize employers and workers as important tools that can be used to eliminate occupational hazards and risk in the work place [3]. OSH also proffers occupational risk preventive measures as well as enhances worker’s productivity. In the agricultural sector, it is postulated that OSH issues exist because of the hazards that are present, ignorance of job hazards, illiteracy, and/or non-existent or inadequate training [4]. According to Demirbas and colleagues, the knowledge level of farmers about OHS hazards and its perceived risks, attitude and behaviour towards job safety are crucial during farming activities [5]. Most farms lack documented OSH policy since they do not operate in an organizational context. Cooper reported that farmers in England have unfavourable attitude towards OSH issues despite being aware of the potential risks associated with their jobs [6]. Until 2000, the agriculture sector was one of the largest labor force globally [7] and with its use of wide range of working tools, livestock, plants and human labor, the agriculture sector workers encounter high occupational risk.

The ILO estimates that more than 170,000 deaths are associated with agricultural work annually [8]; and the sector is described as the most hazardous occupational industry globally [9]. In 2015, the agricultural fatal work injury rate was 22.8 per 100,000 full-time equivalent workers - nearly seven times the all-worker fatal injury rate of 3.4 per 100,000 full-time equivalent worker s[10]. While this statistics is high, it has been reported that the rate of nonfatal occupational injuries in agriculture is largely underestimated [11]. The growing occupational accidents within the agriculture sector calls for the thorough implementation of OSH guidelines. OSH management practices can be expensive but its outcomes can influence workers’ productivity, and hence the profitability and competitiveness of a company. In Sierra Leone like in many other countries with OSH policy, there is a missing link between human resources management and OSH for workers in many companies. This missing link is due primarily to the paucity of research data linking human resources management practices, work place improvement and safety measures. In this study we assessed the effectiveness of the OSH management practices being implemented by Goldtree (SL) Limited Company which is operating in eastern Sierra Leone. We also identified those factors that are responsible for workplace-related accidents or injuries at the company and examined both employees and employer’s perceptions of OHS management practices.

## Methods

### Research settings

Goldtree (SL) Limited Company is a Sierra Leone registered international palm kernel oil producing and marketing company located in Daru in eastern Sierra Leone. The palm kernel oil mill site is located about 370 km from Freetown and about 60 km northeast of Kenema. The company has its plantations located in the following five (5) key locations within Jawei and Malema chiefdoms of Kailahun district; Daru, Tovaima, Lower Jawei, Dambala and Kpangiema. Goldtree (SL) Limited Company is jointly owned by the African Agriculture Fund (AAF), the Finnish Fund for Industrial Cooperation (Finnfund) and the Planting Naturals and currently employs annually on the average permanent and part timers 150 staffs.

### Study subjects and ethics review

This is a cross sectional study in which we administered questionnaires we had designed (Supplemental files) to 100 employees who were attached to various locations and departments at Goldtree (SL) Limited Company. The staffs interviewed in this study were both permanent and contracted employees of the company. Interviews were conducted and questionnaires administered to amass valid qualitative information. To check for accuracy, completeness of data and ensure quality, the questionnaires and interview guide was serially numbered. The Sierra Leone Ethics and Scientific Review Committee (Opinion Date March 29, 2017) and the Goldtree (SL) Limited Company provided ethical clearances and approval respectively for this study. The employees who took part in this study also assented by signing the informed consent statement. The R software data analytical programme (version 3.3.10) and Microsoft excel were used to analyze the data obtained.

## Results

### Study subjects

**Fig. 1:**
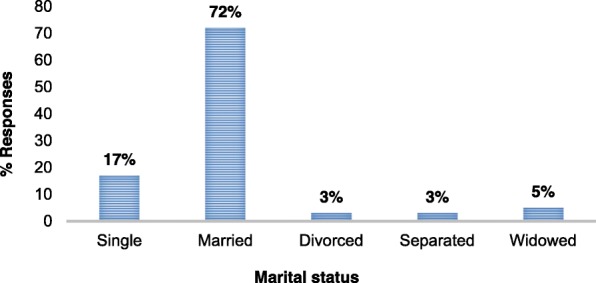
Distribution of workers of Goldtree (SL) Limited Company during the study period based on their marital status

More (71%) men than women took part in this study; majority (64%) of the workers interviewed were below 35 years, few (12%) were between 40 and 50 years and above. Majority (72%) of the workers were married; 17% were single, 3% divorced, 3% separated and 5% were widowed (Fig. 1).

**Fig. 2:**
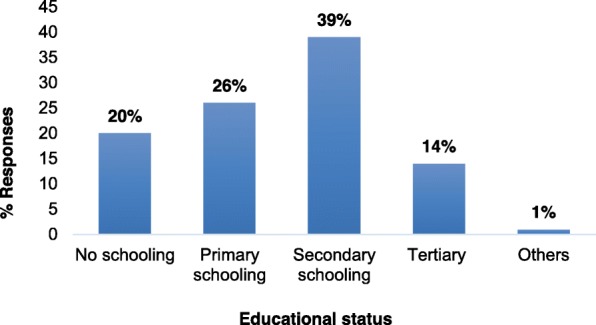
Distribution of workers of Goldtree (SL) Limited Company during the study period based on their educational status

Only 14% of the workers had tertiary level education; 39% had secondary level education, while 26% had primary level education (Fig. 2).

### Length of service of workers

**Fig. 3:**
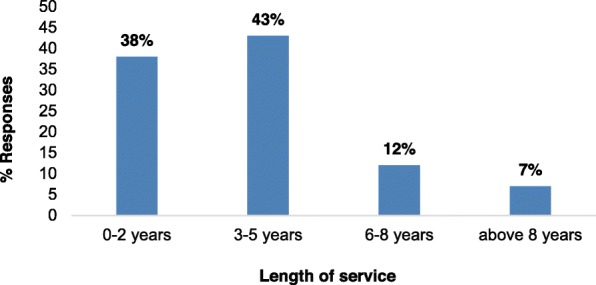
Distribution of workers of Goldtree (SL) Limited Company during the study period based on their length of service at the company

There was an inverse correlation between the duration spent working at Goldtree (SL) Limited Company and the number of workers for that duration of time spent at the company; 43% of the workers had spent 3–5 years at their various departments; 12% had spent 6–8 years, while 7% had spent more than 8 years at their department (Fig. 3).

### Workplace accidents, injuries and reporting

Majority (92.0%) of the workers who encountered workplace accidents or injuries reported them immediately they occurred. Of those workers (*n* = 39, 39%) who encountered workplace accidents or injuries, 38.5% attributed their accident to the lack of adequate safety gears, 10.4% to inadequate training, 12.8% to ignorance about OHS matters, 10.3% to workers non-compliance to OHS regulations and carelessness, 2.6% to improper housekeeping of working tools, and 25.4% attributed their accidents or injuries to a combination of all of the above factors.

Majority (38.4%) of the occupational accidents at Goldtree (SL) Limited Company were associated with lack of adequate safety gears (Table 1).

**Table 1:** Factors responsible for workplace accidents or injuries among workers at Goldtree (SL) Limited Company during the period under review

Factors associated with workplace accidents and injuries	Responses	Percentage (%)
Lack of adequate safety gears	15	38.4
Lack of adequate training	4	10.3
Worker’s ignorant on OHS matters	5	12.8
Worker’s non-compliance and carelessness	4	10.3
Poor housekeeping and unsafe workplace	1	2.6
All of the above	10	25.6
Total	39	100.0

### Likelihood of a workplace accident

We used logistic multivariate regression analysis to determine the odds of occupational accidents or injuries occurring at Goldtree (SL) Limited Company based on industrial and human factors. Workers who have spent 3–5 years on the job (AOR = 2.22, 95% CI = 1.64–7.04, *p* = < 0.05), mill department workers (AOR = 3.31, 95% CI = 2.96–4.07, *p* = < 0.05), those workers that believed that the management is committed to addressing health and safety issues (AOR = 2.49, 95% CI = 1.14–3.19, *p* = < 0.05), believed that their working tools were in excellent condition (AOR = 3.13, 95% CI = 1.99–4.66, *p* = < 0.05), or believed that they have rights and responsibilities of ensuring an effective workplace health and safety management including compliance to OHS measures, the right to refuse unsafe work and reporting occupational accident to the company’s management (AOR =1.11, 95% CI = 1.07–3.54, *p* = < 0.05) have decreased odds of experiencing occupational accidents. Additionally, holding other covariate in the model constant, the odds of experiencing occupational accidents was elevated among workers who believed that management’s commitment to providing the appropriate personnel to handle health and safety issues was a challenge (AOR =7.57, 95% CI = 1.61–9.78, *p* = < 0.05) in the company (Table 2).

**Table 2:** Multivariate logistic regression analysis of factors associated with occupational accident Goldtree (SL) Limited Company

Variables	OR	*p* - value	AOR	95% CI	*p* - value
Mill Department	0.71	0.1	3.31	2.96–4.07	0.04
Length of work between 3 and 5 years	0.45	0.01	2.22	1.64–7.04	0.03
Company’s commitment on OHS	0.75	0.09	2.49	1.14–3.19	0.01
Excellent working logistics	0.51	0.02	3.13	1.99–4.66	0.01
Compliance with OHS measures	0.66	0.01	1.11	1.07–3.54	0.03
Availability of OHS personnel	0.66	0.01	7.57	1.61–9.78	0.05

### Distribution of workplace accident with length of service

**Fig. 4:**
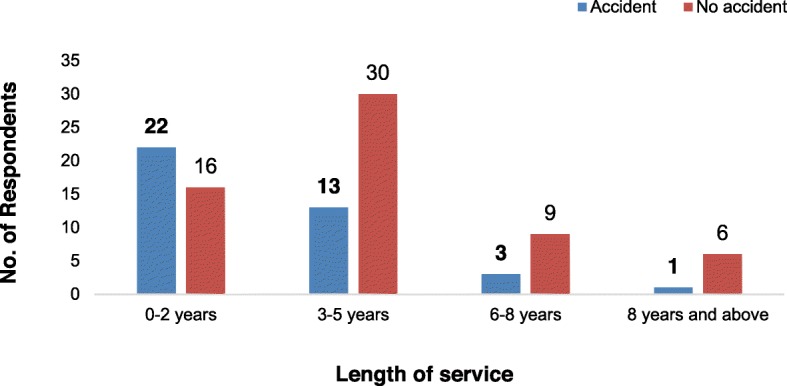
Distribution of workers of Goldtree (SL) Limited Company during the study period based on their length of service at the company and accident occurrence

There is a positive association between the number of workplace accidents or injuries among workers and the length of service of workers (Fig. 4). Of the 39 (39%) workers who reported workplace accidents or injuries, majority (56.4%) have been working at Goldtree (SL) Limited Company for at least 2 years; 33% have been working for 3–5 years, 7.7% have been working for 6–8 years while 2.6% have been working for more than 8 years (X^2^ = 9.8778, *p*-value = 0.02).

## Discussion

OSH management is important in ensuring the health and safety of employees in the workplace. Failure to adhere to OSH practices can have serious consequences. We reported that 39% of the workers interviewed have experienced some form of a workplace accident or injury since their employment at Goldtree (SL) Limited Company. Majority (38.5%) of those who suffered from these accidents or injuries attributed it to the lack of adequate safety gears. Additionally, 92% of those who suffered a workplace accident or injury reported to the company. This is because failing to report a workplace accident or injury to the company’s management will lead to the imposition of due penalties and sometimes can hinder the process of accessing medical service.

We were able to show a decreased odds of experiencing occupational accidents among workers who have spent between 3 and 5 years on the job, workers working in the mill department, workers who believed management is committed to addressing health and safety issues, workers who believed that their working tools were in excellent condition, as well as workers who agreed that they have rights and responsibilities for an effective workplace health and safety system compared to other workers who responded otherwise. However, there was an elevated odds of experiencing occupational accidents among workers who believed that management’s commitment to provide the appropriate personnel to handle health and safety issues was a challenge. We also reported that the non-provision safety gears and equipment was the leading cause of workplace accidents or injuries at the company.

The agroindustry is a physically demanding sector with high potential risks of occupational accident and injuries that are largely preventable [12] through the development of safety OHS programs and intervention systems. Our agro-industrial work-related accident and injuries amongst workers is similar to that reported in other studies [13–15]. Chercos and colleagues reported 36.7% work-related accidents and injuries in their study of agricultural workers in Ethiopia. Our finding which linked work environmental factors, work site, length of time on the job, compliance to OHS policy, availability of work logistics to work-related accidents and injuries is also in agreement with several previous studies [16–21].

## Conclusion

Our study was able to discover key issues relating to occupational accident especially in the agro-industry. We are recommending that employers’ management should ensure the timely purchase and distribution of adequate safety gears and equipment to all workers as well enforce strict adherence and compliance for their use. We are also calling on management to organize regular OSH workshops, job training and seminars for their employees so as to provide them with regular updates on OSH issues. Our findings underpinned the importance of conducting periodic monitoring and on-the-spot checks on companies to ascertain whether they are providing adequate safety gears and materials necessary for an effective workplace health and safety system.

## Supplementary information

**Additional file 1.** The supplemental material document contains the survey questionnaire used in this study.

## Data Availability

The dataset generated and analyzed during this study are not public but can be accessed if sufficient request is made to the corresponding author.

## Abbreviations

AOR: Adjusted Odds Ratio
ILO: International Labour Organisation
OSH: Occupational Safety and Health
SL: Sierra Leone
CI: Confidence Interval
