# Supplementary material for: Agro-industrial accidents linked to length of service, operation site and confidence in employer adherence to safety rules
Source: BMC Public Health. 2020 Apr 30;20:591. doi: 10.1186/s12889-020-08733-2 (PMC7191814; doi:10.1186/s12889-020-08733-2)
Supplement: Supplementary file 1 — Additional file 1. The supplemental material document contains the survey questionnaire used in this study. [file 12889_2020_8733_MOESM1_ESM.docx]

**APPENDIX 1**

**Research Questionnaire**

Hello! I am a Masters’ student at Njala University pursuing Master of Public Health degree. I am conducting a student research. Please, provide your responses for this questionnaire for the study entitled: ***Assessing the Effectiveness of Occupational Health and Safety Management Practices at Goldtree Oil Palm Company***. Please note, since this research is for academic purpose, all information provided will be treated with confidentiality and your anonymity is assured.

1. **Biographic Characteristics**
2. Gender: 1= Male [ ] 2= Female [ ]
3. What is your age group? a) Under 25 ( ) b) between 26-35 ( ) c) 36-45 ( ) d) 46-55 ( ) e) 56 + ( )
4. Marital status: 1=Single [ ] 2=Married [ ] 3=Divorced [ ] 4=Separated [ ] 5= Widowed [ ]
5. Educational level: 0=No schooling [ ] 1=Primary education [ ] 2=Secondary education [ ] 3=Tertiary [ ] 4=Others (Please specify) [ ]
6. Which department do you work? _____________________________
7. How long have you worked at Goldtree? a) 0 – 2yrs. [ ] b) 3 – 5yrs. [ ] c) 6 – 8yrs. [ ] d) 8 yrs.+ [ ]
8. In what capacity have you been working? ___________________________
9. **Factors Responsible For Workplace Accidents and General Workplace Safety Management**

*The following are multiple-choice questions. More than one option may be correct. Please tick in the boxes the correct response (s)*

8. Have you ever suffered any workplace accident/injuries since you were employed? YES [ ] NO [ ]

9. If yes, what do you think are the major factors responsible for workplace accidents at Goldtree?

a) Lack of adequate personal protective clothing and equipment ( )

b) Lack of adequate training on workplace health and safety and on the job ( )

c) Workers ignorance on occupational health and safety matters ( )

d) Employee’s non-compliance and careless work attitude ( )

e) Poor Housekeeping and unsafe work environment ( )

f) All of the above ( )

g) Others (please specify) _______________________________________________________

10. If yes, did you report the accident to the appropriate authorities at Goldtree? a) YES ( ) b) NO ( )

11. If yes, what was the immediate action taken by the management? a) First aid was given [ ] b) taken to hospital [ ] c) no action taken [ ]

12. How would you rate your working environment? FAIR [ ] GOOD [ ] EXCELLENT [ ]

13. How would you rate the efficiency of your working Machines/Tools/ Equipment? (If any)

FAIR [ ] GOOD [ ] EXCELLENT [ ]

14. Do you have personal protective equipment (PPE)? YES [ ] NO [ ]

15. If yes, do you use them correctly on the job? (Researcher’s observation) YES [ ] NO [ ]

16. If your answer is No to 14, please state your reason/s: _______________________________

17. Does Goldtree provide adequate workplace health and safety training? YES [ ] NO [ ]

**C) Respondents’ Perceptions on Health and Safety Management Practices**

*The following are multiple-choice questions. More than one option may be correct. Please tick below to express your opinion on the following questions:*

18. What do you understand by occupational health and safety (OHS)?

a) Employees’ welfare ( ) b) Employers’ welfare ( ) c) Both employer and employee welfare ( ) d) Employers, employees and third party welfare ( ) e) others (please specify) ___________________

19. Employees are required to put on protective clothing in the performance of their duties

a) TRUE ( ) b) FALSE ( ) c) NOT SURE ( )

20. Both employers and employees have responsibilities and rights for effective work place health and safety management. a) YES ( ) b) NO ( ) c) NOT SURE ( )

21. If yes, what are some of the responsibilities and rights of employees?

a) Compliance with safety measures/procedures put by management ( ) b) Wearing personal protective clothing ( ) c) The right to refuse unsafe work ( ) d) All of the above ( )

22. If yes, what are some of the responsibilities and rights of employers?

a) Provide safe place of work ( ) b) Provide safety equipment and procedures ( ) c) Provide training on health and safety ( ) d) Provide first aid and medical care for employees ( ) e) All of the above ( )

23. What are some of the safety measures put in place in your department?

a) Use of personal protective equipment and complying with safe work procedures ( )

b) Provision of adequate health and safety, and on the job training ( )

c) Use of standard operating procedures and following good work practices ( )

d) Proper housekeeping and safe disposal of waste materials ( )

e) Regular monitoring on health and safety standards to ensure compliance ( )

f) Prompt reporting of accidents/injuries/near misses ( )

g) Conducting fire drills and workplace emergency evacuation procedures ( )

h) All of the above ( )

i) Others (Please specify) ___________________________________________________

24. Indicate how satisfied you are with the current health and safety measures put in place at Goldtree

a) Very satisfied ( ) b) Satisfied ( ) c) Dissatisfied ( ) d) Very Dissatisfied ( )

25. Who is ultimately responsible for your health and safety in the performance of your job?

a) Management ( ) b) Line Manager ( ) c) HSE Manager ( ) e) your supervisor ( ) d) Yourself ( )

e) All of the above ( )

26. What should Goldtree management do to further improve health and safety practices?

a) Provision of adequate safety gears on timely basis b) Ensure staff training and compliance ( ) c) Review workplace health and safety practices ( ) d) Ensure periodic on the job training ( )

e) Improve on good housekeeping and sanitation ( ) f) Create the environment for staff to freely report on occupational health and safety ( ) i) others (Please specify) ______________________________

1. **Compliance with Occupational Health and Safety Standards**

*Please indicate your level of agreement or disagreement with the issues below:*

27. Goldtree ensures that employees are not subjected to unreasonable risks in the workplace. Strongly Agree [ ] Agree [ ] Disagree [ ] Strongly Disagree [ ] Neutral [ ]

28. Goldtree encourages workers to report work related accidents and injuries

Strongly Agree [ ] Agree [ ] Disagree [ ] Strongly Disagree [ ] Neutral [ ]

29. Goldtree conducts regular inspections to assess health and safety in the workplace

Strongly Agree [ ] Agree [ ] Disagree [ ] Strongly Disagree [ ] Neutral [ ]

30. Goldtree management responds quickly to workplace health and safety concerns

Strongly Agree [ ] Agree [ ] Disagree [ ] Strongly Disagree [ ] Neutral [ ]

31. Goldtree has person/s trained to manage health and safety and incident investigation procedure Strongly Agree [ ] Agree [ ] Disagree [ ] Strongly Disagree [ ] Neutral [ ]

32. Goldtree provides appropriate remedy for addressing accidents occurrence

Strongly Agree [ ] Agree [ ] Disagree [ ] Strongly Disagree [ ] Neutral [ ]

1. **Challenges for Non- Compliance with Occupational Health and Safety Standards**

*Please indicate your agreement or disagreement with the issues below:*

33. The provision of personal protective equipment has been an extra cost burden on the company

Strongly Agree [ ] Agree [ ] Disagree [ ] Strongly Disagree [ ] Neutral [ ]

34. The huge working population with low literacy rate and ignorance on health and safety is a challenge. Strongly Agree [ ] Agree [ ] Disagree [ ] Strongly Disagree [ ] Neutral [ ]

35. Getting the right personnel to help in promoting health and safety is a challenge

Strongly Agree [ ] Agree [ ] Disagree [ ] Strongly Disagree [ ] Neutral [ ]

36. Getting management to be committed to health and safety is a challenge

Strongly Agree [ ] Agree [ ] Disagree [ ] Strongly Disagree [ ] Neutral [ ]

37. Workers refusal to report workplace accidents/injury for fear of being sacked is a challenge Strongly Agree [ ] Agree [ ] Disagree [ ] Strongly Disagree [ ] Neutral [ ]

38. Workers refusal to wear PPE and follow right work procedures is a challenge

Strongly Agree [ ] Agree [ ] Disagree [ ] Strongly Disagree [ ] Neutral [ ]

39. Negative attitude and carelessness of some workers on health and safety is a challenge

Strongly Agree [ ] Agree [ ] Disagree [ ] Strongly Disagree [ ] Neutral [ ]

40. Cost involve in training employees and providing the required health and safety resources is a challenge. Strongly Agree [ ] Agree [ ] Disagree [ ] Strongly Disagree [ ] Neutral [ ]

1. **Measures Adopted to Improve OHS and Compliance with International Best Standards**

*Please indicate your agreement or disagreement with the issues below:*

41. Goldtree provides safe working environment and conditions for workers.

Strongly Agree [ ] Agree [ ] Disagree [ ] Strongly Disagree [ ] Neutral [ ]

42. Goldtree provides adequate personal protective equipment for workers safety

Strongly Agree [ ] Agree [ ] Disagree [ ] Strongly Disagree [ ]

43. Goldtree provides adequate workplace health and safety trainings for workers

Strongly Agree [ ] Agree [ ] Disagree [ ] Strongly Disagree [ ] Neutral [ ]

44. Goldtree provides adequate on the job training and assess safe job performance

Strongly Agree [ ] Agree [ ] Disagree [ ] Strongly Disagree [ ] Neutral [ ]

45. Goldtree provides standard operating procedures (SOP) for safe job performance

Strongly Agree [ ] Agree [ ] Disagree [ ] Strongly Disagree [ ] Neutral [ ]

46. Goldtree ensures safety materials provided are used correctly at the workplace

Strongly Agree [ ] Agree [ ] Disagree [ ] Strongly Disagree [ ] Neutral [ ]

47. Goldtree provides notices and key information on health and safety matters

Strongly Agree [ ] Agree [ ] Disagree [ ] Strongly Disagree [ ] Neutral [ ]

48. Co-workers at Goldtree ensure their safety and the safety of their co-workers

Strongly Agree [ ] Agree [ ] Disagree [ ] Strongly Disagree [ ] Neutral [ ]

49. Goldtree provides first aid services and medical services for workers

Strongly Agree [ ] Agree [ ] Disagree [ ] Strongly Disagree [ ] Neutral [ ]

50. Goldtree ensures investigations of work related accidents and injuries

Strongly Agree [ ] Agree [ ] Disagree [ ] Strongly Disagree [ ] Neutral [ ]

51. Goldtree institutes discipline and due penalties for non-compliance

Strongly Agree [ ] Agree [ ] Disagree [ ] Strongly Disagree [ ] Neutral [ ]

52. Goldtree provides incentives for workers who comply with health and safety standards

Strongly Agree [ ] Agree [ ] Disagree [ ] Strongly Disagree [ ] Neutral [ ]

53. Does Goldtree provides adequate information and training on international best standards (e.g. RSPO/ Organic Farming or Fairtrade etc.) a) YES ( ) b) NO ( ) c) NOT SURE ( )

54. Did you know that for Organic farming of oil palm, you may NOT use inorganic chemicals such as NPK, Glyphosate?   a) YES ( ) b) NO ( ) c) NOT SURE ( )

55. Did you know that for Organic farming of oil palm, you may NOT use fire for land preparation and weeding?   a) YES ( ) b) NO ( ) c) NOT SURE ( )

56. Did you know that for Organic farming of oil palm, you MUST recycle organic materials such as old leaves, empty bunches on the field?  a) YES ( ) b) NO ( ) c) NOT SURE ( )

57. Did you know that for Organic farming of oil palm, you MUST clean your farm from inorganic waste (plastics, metal, glass, etc.)?   a) YES ( ) b) NO ( ) c) NOT SURE ( )

58. Did you know that RSPO stands for Roundtable on Sustainable Palm Oil?  a) YES ( ) b) NO ( ) c) NOT SURE ( )

59. Did you know that RSPO strictly forbids the employment of children below 18 years old?

a) YES ( ) b) NO ( ) c) NOT SURE ( )

60. Did you know that RSPO requires Goldtree to provide their workers with appropriate personal protective equipment (e.g.; rain boots, helmets) and training?  a) YES ( ) b) NO ( ) c) NOT SURE ( )

61. Did you know that RSPO requires Goldtree to follow strict procedures (stakeholder involvement, FPIC engagement, HCV assessments, etc.) for new plantings?   a) YES ( ) b) NO ( ) c) NOT SURE ( )

62. As an employee, how would you assess both RSPO and Organic Farming Principles as part of Goldtree’s operational standards? FAIR [ ] GOOD [ ] EXCELLENT [ ] NOT SURE [ ]

**Signed: _______________________**

Emmanuel T. Koroma
